# CD1d Expression and Invariant NKT Cell Responses in Herpesvirus Infections

**DOI:** 10.3389/fimmu.2015.00312

**Published:** 2015-06-25

**Authors:** Brian K. Chung, John J. Priatel, Rusung Tan

**Affiliations:** ^1^NIHR Birmingham Liver Biomedical Research Unit, Centre for Liver Research, University of Birmingham, Birmingham, UK; ^2^Institute of Clinical Medicine, Faculty of Medicine, University of Oslo, Oslo, Norway; ^3^Department of Pathology and Laboratory Medicine, University of British Columbia, Vancouver, BC, Canada; ^4^Department of Pathology, Sidra Medical and Research Center, Doha, Qatar

**Keywords:** iNKT cells, CD1d, herpesvirus, viral immunity, immunotherapy

## Abstract

Invariant natural killer T (iNKT) cells are a highly conserved subset of unconventional T lymphocytes that express a canonical, semi-invariant T cell receptor and surface markers shared with the natural killer cell lineage. iNKT cells recognize exogenous and endogenous glycolipid antigens restricted by non-polymorphic CD1d molecules, and are highly responsive to the prototypical agonist, α-galactosylceramide. Upon activation, iNKT cells rapidly coordinate signaling between innate and adaptive immune cells through the secretion of proinflammatory cytokines, leading to the maturation of antigen-presenting cells, and expansion of antigen-specific CD4+ and CD8+ T cells. Because of their potent immunoregulatory properties, iNKT cells have been extensively studied and are known to play a pivotal role in mediating immune responses against microbial pathogens including viruses. Here, we review evidence that herpesviruses manipulate CD1d expression to escape iNKT cell surveillance and establish lifelong latency in humans. Collectively, published findings suggest that iNKT cells play critical roles in anti-herpesvirus immune responses and could be harnessed therapeutically to limit viral infection and viral-associated disease.

## Introduction

Herpesviridae is a family of large DNA viruses that contain between 100 and 200 genes within an icosahedral capsid composed of viral proteins, mRNAs, and a lipid bilayer envelope (1). In humans, herpesviruses frequently infect both immunocompetent and immunocompromised hosts, with high-prevalence rates ranging from 60 to 90% in the adult population (2, 3). Common human herpesviruses include herpes simplex type 1 (HSV-1) and type 2 (HSV-2), varicella zoster virus (VZV), human cytomegalovirus (HCMV), Epstein–Barr virus (EBV), human herpesvirus 6 (HHV-6), and Kaposi’s sarcoma-associated herpesvirus (KSHV). Primary infections with herpesviruses are frequently mild or asymptomatic and lead to lifelong viral latency within the host. However, reactivation of viral replication in immunocompromised individuals often leads to life-threatening infections and malignancies (4).

Host immune responses are critical for restraining and abrogating viral replication, controlling viral load, and limiting disease severity (5–10). For example, HSV and HCMV infections in immunocompetent individuals trigger a rapid expansion of natural killer (NK) cells and virus-specific cytotoxic T lymphocytes (CTL) that are important for eliminating infected cells (3, 11, 12). In response, herpesviruses have evolved sophisticated strategies to evade NK cell and CTL recognition that allow herpesviruses to achieve lifelong survival. In the case of CTL, whose T cell receptor (TCR) bind virus peptide–MHC class I complexes on the infected cell surface, herpesviruses have been shown to disrupt many steps of MHC class I presentation, including the transfer of cytosolic peptides into the ER, the loading of peptides onto newly synthesized MHC complexes, and the trafficking of MHC–peptide molecules from the cytosol to the plasma membrane (13, 14). In contrast to CTL, NK cells lack TCR and respond to reduced MHC class I expression induced by herpesvirus infection (13, 15). Inhibitory NK surface markers, such as killer cell immunoglobulin-like receptors (KIR), leukocyte immunoglobulin-like receptors (LIR), and CD94/NKG2 (15, 16), monitor the expression of self-MHC class I and prevent the activation of NK cells. Herpesvirus infections that downregulate MHC class I surface expression in order to evade CTL are more susceptible to NK cells and hence, some herpesviruses also express viral homologs of MHC class I to escape NK cell detection (14, 17). The existence of these back and forth CTL and NK cell evasion strategies underscores their presumed importance in controlling herpesvirus infection and provides a rationale for why multiple immune subsets are necessary to effectively combat herpesvirses.

Natural killer T (NKT) cells are a unique group of CD1d-restricted innate-like lymphocytes and patients deficient in NKT cells develop severe and fatal herpesvirus infections (18–24). These findings, in concert with observations showing that herpesviruses downregulate surface expression of CD1d (25, 26), suggest an important role for NKT cells in the immune response to herpesviruses. NKT cells are distinct from NK cells as they express TCR, but unlike CTL, NKT cells emigrate from the thymus primed to respond and aid in early anti-viral defenses. In this review, we focus on invariant natural killer T (iNKT) cells, a population of NKT cells, which recognize the exogenous lipid antigen, α-GalCer (27). We highlight the role of iNKT cells in herpesvirus infections and the significance of CD1d expression in controlling herpesvirus replication.

## iNKT Cells – Unconventional T Lymphocytes

Invariant natural killer T cells are a subset of T lymphocytes that express a canonical, semi-invariant TCR and surface markers typically found on NK cells and activated CTL (28–30). iNKT cells are positively selected in the thymus by the non-polymorphic glycoprotein, CD1d, and recognize CD1d-restricted glycolipid antigens presented by antigen-presenting cells (APC) in the periphery. Human iNKT cells are CD4+, CD8+ or CD4−CD8− and bear a Vα24–Jα18 TCR rearrangement that preferentially associates with Vβ11 (31, 32). In mice, CD8+ iNKT cells are rare and the majority express a Vα14–Jα18 TCR α-chain paired with Vβ8, Vβ7, or Vβ2 (33, 34). Human and mouse iNKT cells both display an effector memory phenotype (CXCR3+, CXCR4+, CD44+, CD69+, CD161+ in humans, NK1.1+ in mice) (35, 36) and are strongly activated by α-GalCer, a non-mammalian glycosphingolipid originally isolated from a marine sea sponge (27). In contrast to iNKT cells (type I), variant NKT cells (diverse or type II) are unresponsive to α-GalCer, and react to sulfatide and phospholipid antigens (37). Type II NKT cells are largely excluded from this review as much less is known about their role in viral infection.

## iNKT Cell Activation in Viral Infection

In recent years, evidence from multiple clinical and animal studies suggest that iNKT cells enhance the control of herpesvirus replication (18–24, 35, 38–41) (Table 1). However, the identity of the lipid antigen(s) that presumably drive iNKT cell activation remains elusive. By contrast, several bacteria-derived lipid antigens containing α-linked glycans similar to α-GalCer have been reported [α-glucuronosylceramide and α-galacturonosylceramide (42–44), α-galatcosydiacylglycerol (45), and α-glucosyldiaglyercerol (46) from *Streptococcus pneumoniae*, *Sphingomonas* *paucimobilis*, and *Borrelia burgedorferi*, respectively]. Unlike bacteria, herpesviruses do not express virus-specific lipids; therefore, in the absence of pathogen-derived antigens, iNKT cells likely recognize endogenous self-lipids presented by CD1d (47). Supporting this assumption are several lines of evidence showing that CD1d is required to activate iNKT cells following human herpesvirus infection (25, 48–53). Moreover, hepatitis B infection has been shown to induce the expression of endogenous lipid antigens (lysophospholipids) in human and mouse hepatocytes (54), suggesting that herpesvirus infection may trigger the presentation of analogous self-lipids on CD1d.

**Table 1:** **Effect of human herpesvirus infection on CD1d expression and iNKT cells**.

			iNKT cell deficiency			
Virus	Human CD1d expression	Mechanisms	Mouse	Human	α-GalCer	References
HSV-1	↑ (Low-viral dose)	Glycoprotein B (gB); serine–threonine kinase, US3	↑ Viral titer	–	–	(48, 51, 55–59)
	↓ (High-viral dose)					
HSV-2	–	–	↑ Viral titer	–	↑ Protection	(49, 60–65)
			↑ Mortality			
VZV	–	–		↑ Disease	–	(23, 40)
EBV	↓	↓ CD1d transcription	–	↑ Viral titer		(18–21, 24, 53, 66–71)
				↑ Disease		
HHV-6A/B	–	–	–	–	–	–
HHV-7	–	–	–	–	–	–
CMV	↓	glycoprotein US2	↑ Viral titer		↓ Viral titer	(52, 72–77)
			↑ Mortality			
KSHV	↓	Modulator of immune recognition-1 and -2 (K3 and K5)	–	–	–	(25, 78, 79)

*HSV-1, herpes simplex virus-1; HSV-2, herpes simplex virus-2; VZV, varicella zoster virus; EBV, Epstein–Barr virus; HHV6A/B, human herpesvirus 6A/B; HHV-7, human herpesvirus-7; CMV, cytomegalovirus; KSHV, Kaposi’s sarcoma-associated herpesvirus; –, unknown*.

Lysophospholipids were identified as endogenous iNKT cell antigens by screening the responsiveness of human iNKT cell clones to synthetic preparations of CD1d-bound ligands (80). Similar filtering procedures were used to identify the glycosphingolipid, β-d-glucopyranosylceramide, as a physiologically relevant self-antigen for iNKT cells (81). Whether these self-antigens are presented by APC during herpesvirus infections is not yet known but the recognition of viral nucleic acids by Toll-like receptors (TLR)-3, -7, and -9 has been shown to induce the synthesis of β-d-glucopyranosylceramide (21), implying that glycosphingolipid antigens may be expressed in herpesvirus infections (81). APC treated with TLR-3, -7, -8, and -9 agonists also enhance transcription of enzymes involved in glycosphinolipid synthesis and the inhibition of these pathways abolishes the reactivity of iNKT cells to TLR-stimulated APC (82, 83). Together, these findings suggest that herpesvirus may activate early iNKT cell responses during infection by inducing the presentation of endogenous lipids antigens on CD1d.

In addition to antigen activation, iNKT cells can react to herpesvirus replication in a CD1d/TCR-independent manner through the actions of proinflammatory cytokines and costimulatory molecules on APC (47). iNKT cells express high levels of IL-12R and are sensitive to IL-12, as well as IL-2, IL-18, and type I IFN released following bacterial (84, 85) and murine cytomegalovirus (MCMV) infection (72, 86). iNKT cells also respond to IL-23 and IL-25 (87, 88), and stimulation by these cytokines induces IL-17 production and amplify inflammatory anti-viral responses (89, 90). Thus, the activation of iNKT cells during herpesvirus infections may involve two pathways; TCR signaling provided by the recognition of lipid antigen(s) and antigen-independent stimuli supplied via cytokines and co-stimulation molecules.

T cell receptor-dependent and -independent activation of iNKT cells can both elicit the substantial release of cytokines and chemokines, including IFN-γ, TNF-α, TNF-β, GM-CSF, IL-2, IL-4, IL-5, IL-6, IL-10, IL-13, IL-17, IL-21, CCL3/MIP-1α, CCL4/MIP-1β, CCL5/RANTES, and eotaxins (91, 92). IFN-γ, TNF-α, and TNF- β are known to have direct inhibitory effects on viral replication and GM-CSF, IL-2, IL-4, IL-5, IL-6, IL-10, IL-13, IL-17, IL-21, CCL3/MIP-1α, CCL4/MIP-1β, CCL5/RANTES activate APC, NK cells, CD4+, and CD8+ T lymphocytes, and promote iNKT cells to migrate to sites of inflammation (36, 92). The early production of cytokines and chemokines by iNKT cells may boost the regulation of anti-herpesvirus defenses by triggering the activation of innate and adaptive immune responses. Further work is necessary to substantiate the production and effect of these cytokines and chemokines *in vivo* as the majority of these molecules are secreted by iNKT cells only after powerful TCR stimulation by α-GalCer and have not been directly assessed during herpesvirus infections.

Along with their potent cytokine abilities, activated iNKT cells can also kill target cells through their expression of perforin/granzyme, TRAIL, and FasL (91). Additional assessment is also required to fully delineate the importance of iNKT cell cytotoxicity in herpesvirus infections but B cells transformed by EBV are susceptible to iNKT-mediated cytolysis *in vitro* (53), suggesting that iNKT cells may directly prevent the proliferation of virus-transformed cells.

### Herpes simplex virus-1

Herpes simplex virus-1 is an α-herpesvirus that infects mucocutaneous epithelium and establishes latency in sensory ganglia (2). HSV-1 is commonly associated with oral and ocular lesions. However, genital HSV-1 infections now account for over half of genital herpes episodes in North American and European countries (93–96). Studies in HSV-1 murine models support a role for iNKT cells in controlling herpesvirus infection: CD1d- and Jα18-deficient mice infected with HSV-1 experience higher viral loads and morbidity compared to wild-type littermates (48). iNKT cells may be dispensable in some strains of HSV-1 infection (55) but help control HSV-1 strains that persist in sensory neurons indicating that iNKT cells may be important for restricting the reactivation of HSV-1 (56).

**Figure 1:**
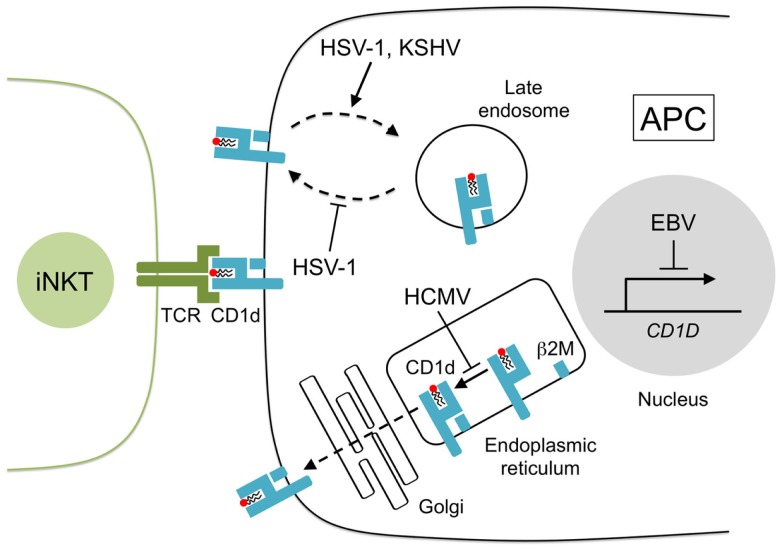
**CD1d presentation is disrupted by human herpesviruses**. Lipids are loaded onto newly synthesized CD1d heavy chains and arranged with β2-microglobulin (β2M) in the endoplasmic reticulum. CD1d–lipid complexes are transported to the cell surface through the Golgi network by exocytosis. CD1d–lipid molecules are recycled from the cell surface by endocytosis and CD1d ligands are exchanged in the late endosome. Herpesviruses inhibit CD1d presentation to iNKT cells in several ways: HCMV glycoprotein US2 interacts with CD1d in the endoplasmic reticulum reducing iNKT cell activity, HSV-1 glycoprotein B, and serine–threonine kinase US3 remove CD1d from the cell surface and prevent its return to the surface, KSHV modulator of immune recognition-1 and -2 (also known as K3 and K5) downregulate surface CD1d by sequestering its expression to the late endosomes, and EBV transformation of B cells suppresses CD1d transcription.

A role for iNKT cells in HSV-1 infection is also supported by observations that HSV-1 alters CD1d presentation, which implies that HSV-1 may modulate CD1d expression to evade iNKT cell recognition. While low-dose HSV-1 infection in human myeloid dendritic cells (DC) increases surface CD1d expression (51, 57), infection with high-viral titers triggers the rapid re-distribution of surface CD1d molecules to the limiting membrane of lysosomes and the *trans*-Golgi network (Figure 1), an action mediated by HSV-1 glycoprotein B (gB) and the viral serine–threonine kinase, US3, which inhibits the activation of iNKT cells (26, 58). HSV-1 may also suppress the stimulation of iNKT cells in a CD1d-independent manner as HSV-1 infection in keratinocytes has no effect on CD1d but still impairs iNKT cell activation through an undetermined contact-dependent mechanism (59). These findings support the participation of iNKT cells in anti-HSV-1 responses as HSV-1 appears to have evolved specific mechanisms that suppress iNKT cell function.

### Herpes simplex virus-2

Herpes simplex virus-2, also an α-herpesvirus, shares significant DNA sequence homology with HSV-1 (97) but is more often linked with genital mucocutaneous infections and persistence in innervating sensory neurons than mucocutaneous epithelium (98). Vertical transmission of HSV-2 by infected mothers to newborns results in neonatal herpes, a serious disease with high rates of neurological complications and mortality (99, 100).

In mice, iNKT cells appear to strongly influence HSV-2 replication as CD1d-deficient animals are 10-fold more susceptible to severe infection compared to wild-type controls (49). iNKT cells are early producers of IFN-γ in HSV-2 infection (49) and can also secrete large quantities of IL-21 that can trigger NK cell and CTL function (60), reduce infection severity and improve host survival (61). IL-21 production by iNKT cells may be particularly critical in limiting HSV-2 replication at the site of infection as the IL-21R expression is upregulated on vaginal epithelia 1–3 days post-infection, and similar to CD1d-deficient mice, IL-21R-deficient animals have increased viral loads and higher mortality to HSV-2 (61).

Severely reduced iNKT cell numbers and a complete lack of NK cells are also observed in IL-15-deficient mice (62) providing further evidence that iNKT cells play a role in controlling HSV-2 as IL-15-deficient mice display a heightened sensitivity to HSV-2 infection (100-fold) compared to CD1d-deficient mice (49). This finding supports the notion that iNKT cells are important in anti-HSV-2 defenses and that they may act synergistically with NK cells to augment host responses to HSV-2. It must be noted, however, that IL-15 can mediate innate immunity against HSV-2 independently of iNKT cells and NK cells (63), and that IL-15 is released by human peripheral blood mononuclear cells (PBMC) upon HSV-2 infection (64).

iNKT cells could be an effective immunotherapy against HSV-2 as intranasal and intravaginal immunization with α-GalCer and HSV-2 glycoprotein (gD) elicits robust innate immunity, the development of systemic gD-specific antibodies and strong secondary responses to HSV-2 proteins in mice (65). Intravaginal immunization provides complete protection against lethal vaginal HSV-2 infection, which supports further evaluation of α-GalCer as an adjuvant for HSV-2 vaccines.

### Varicella zoster virus

Varicella zoster virus is a neurotropic α-herpesvirus that commonly causes varicella (chicken pox) and subsequently herpes zoster in humans (101). Since its introduction in 1974 (102), live attenuated varicella vaccine has been routinely used worldwide with a wide-safety profile in healthy children although a small number of apparently normal children have been described to develop severe complications such as pneumonitis (23, 40, 103, 104). Immune phenotyping in two of these patients revealed a profound reduction of peripheral blood iNKT cells (23, 40). In the first case, an 11-year-old girl developed a papulovesicular rash and adverse respiratory illness several weeks after receiving varicella vaccine (23). Analysis of her peripheral lymphocytes at 2 and 4 months after her recovery showed a striking lack of iNKT cells and a complete absence of IFN-γ production by her PBMC following α-GalCer stimulation. The second report describes a 6-year-old boy that presented with vesicular rash and life-threatening pneumonitis 3 weeks after varicella vaccination (40). He too had a reduced number of peripheral iNKT cells, but unlike the first patient, α-GalCer elicited an IFN-γ response from his PBMC, albeit approximately two-fold less than controls. IFN-γ production by his conventional T cells was also decreased upon stimulation with a low concentration of the polycolonal T cell mitogen, PHA, suggesting that the patient may have had a global IFN-γ defect in addition to low iNKT cell numbers. CD1d expression on the surface of his APC was undetectable and CD1d RNA levels were approximately two-fold lower compared to controls. This observation raises the interesting possibility that circulating iNKT cell numbers in this patient may have been affected by the absence of CD1d on his APC. These case reports suggest that iNKT cells may be activated during VZV infection and future studies quantifying their activation and expansion following VZV vaccination would help delineate the contribution of iNKT cells to anti-VZV defenses.

### Epstein–barr virus

Epstein–Barr virus is a γ-herpesvirus and primary infection in childhood is generally asymptomatic whereas exposure in adolescence or young adulthood often presents as infectious mononucleosis (IM) (66). EBV is strongly associated with several cancers including nasopharyngeal carcinoma in immunocompetent adults, and a variety of B cell and other malignancies in immunocompromised individuals with AIDS or following transplant immunosuppression (105).

There is extensive evidence that iNKT cells are a critical component of immune responses to EBV, but much of the data are inconclusive or circumstantial because it originates from humans with rare monogenic disorders and clinical case reports. Boys with mutations in the *SH2D1A* gene, which encodes SLAM-associated protein (SAP), have a complete absence of iNKT cells (18–20) and develop X-linked lymphoproliferative disease (XLP) (67, 68), a form of severe and often fatal IM typically triggered by EBV infection (66). It is difficult to ascribe the symptoms of XLP to iNKT cell defects alone because SAP mutations impair iNKT cell development and also disrupt the function of NK cells, CD4+, and CD8+ T cells (69).

Patients with defects in X-linked inhibitor of apoptosis (XIAP) also present with an XLP-like syndrome and have reduced iNKT cell numbers (21). However, the link between iNKT cells and XIAP is unclear given that XIAP-deficient mice have normal numbers of iNKT cells, whereas SAP-deficient mice closely mimic the phenotype of XLP patients and share an impaired development of iNKT cells (70). These findings suggest that patients lacking SAP or XIAP may be susceptible to EBV because of different signaling defects despite exhibiting a similar absence of iNKT cells.

A case report on two sisters who died from an EBV-associated lymphoproliferative disorder resembling XLP strengthens the argument that iNKT cells are involved in the normal control of EBV replication (24). Genetic studies on the two siblings revealed that both sisters had inherited a homozygous mutation in IL-2-inducible T cell kinase (*ITK*) and immune phenotyping revealed a total absence of iNKT cells, a finding that is recapitulated in ITK-deficient mice (71). This study, along with the previous reports in XLP patients, implies genetic mutations that impair iNKT cell development (*SH2D1A*, *XIAP*, *ITK*) may be critical risk factors in determining susceptible to EBV-associated diseases. Additional studies are warranted to clearly elucidate the contribution of iNKT cells in anti-EBV responses and determine if iNKT cells can be targeted for use in EBV vaccines.

iNKT cells may also be involved in the control of EBV-associated cancers. We have shown that the transformation of human B cells into lymphoblastoid cell lines (LCL) rapidly triggers the loss of CD1d transcription and surface expression due to the increased binding of lymphoid enhancer-binding factor 1 (LEF-1) to the CD1d promoter region (53) (Figure 1). LEF-1 is a nuclear protein and dimerizes with β-catenin to suppress CD1d promoter activity (73, 106). Treatment of LCL with the retinoic acid receptor agonist, AM580, prevents the accumulation of LEF-1 at the CD1d promoter, restores the transcription and surface expression of CD1d, and activates human iNKT cell lines to recognize LCL even in the absence of α-GalCer. These findings suggest that EBV transformation may induce the expression of endogenous lipid antigens and that the modulation of the retinoic acid pathway could improve iNKT cell regulation of EBV malignancies.

### Human cytomegalovirus

Human cytomegalovirus is a polytropic β-herpesvirus and the largest member of the herpesvirus family (100). Infection by HCMV is usually asymptomatic but primary and reactivated disease in immunocompromised individuals is associated with significant morbidity and mortality (7, 74). HCMV appears to evade iNKT cell surveillance by expressing the HCMV glycoprotein, US2, which interacts with CD1d (75) and facilitates its proteasomal degradation *in vitro* (76) (Figure 1). The precise contribution of iNKT cells during HCMV infection *in vivo* is less conclusive but murine cytomegalovirus (MCMV) has been widely used as an experimental model for HCMV and in this model, iNKT cells appear to assist early immune responses against MCMV (52, 72, 77) despite an earlier report to the contrary (107). As expected, iNKT cells produce substantial levels of IFN-γ and perforin shortly after MCMV challenge but the addition of TCR blockers or CD1d antibody prior to infection had minimal effect on iNKT cell function (72) indicating that iNKT cell activation by MCMV may be CD1d-independent and could be a consequence of IL-12 production by TLR-9-stimulated APC (86, 108). The relevance of iNKT cells in anti-HCMV defenses requires future clarification as Jα18-deficient mice (specifically lack iNKT cells) show similar mortality rates as wild-type controls after high dose MCMV infection (72, 107). By contrast, CD1d-deficient mice (lack both iNKT cells and type II NKT cells) show an increased MCMV susceptibility (72) suggesting that type II NKT cells may play a larger role than iNKT cells in the regulation of HCMV.

### Kaposi’s sarcoma-associated herpesvirus

KSHV is a γ-herpesvirus that can cause malignancies including Kaposi’s sarcoma, primary effusion lymphoma, and multicentric Castleman’s disease (1, 78, 79). A putative role for iNKT cells in anti-KSV responses was inferred by the finding that KSHV infection of B cells leads to the sequestering of CD1d to the endocytic pathway and a subsequent loss of iNKT recognition (25). CD1d is directed away from the cell surface by the KSHV-encoded ubiquitin ligases, modulator of immune recognition (MIR)-1, and MIR-2 (also known as K3 and K5), which ubiquitinate the cytosolic lysine residues of CD1d and prevent CD1d from recycling to the plasma membrane (25) (Figure 1). MIR-2 also downregulates the expression of the NKG2D ligands, MHC class I-related chain A (MICA), and MICB (109). NKG2D signaling is known to activate iNKT cell function in the absence of TCR stimulation (110); therefore, the loss of NKG2D signaling may represent another mechanism by which KSHV can control iNKT cell activation during infection.

## Conclusion

Mounting evidence supports a significant role for iNKT cells in bridging innate and adaptive immune defenses during herpesvirus infection. Clinical case reports and animal studies demonstrate that iNKT cells may prevent severe and fatal herpesvirus infections (Table 1). Given that herpesviruses interfere with CD1d–iNKT recognition empirically suggests that virus survival and persistence may benefit from the evasion of iNKT cell surveillance.

Significant progress over the last decade has greatly improved our understanding of iNKT cell biology but the precise nature of the CD1d-restricted antigens that activate iNKT cells in herpesvirus infections is still unknown. Discovering the identity of these virus-induced lipid antigens is a priority that will greatly improve the understanding of anti-viral iNKT cell responses *in vivo* and would provide stronger evidence that iNKT cells contribute to anti-herpesvirus defenses. These findings could also assist the development of iNKT cell-based therapies that specifically target pathways that induce the expression of lipid antigens.

Published studies have shown that herpesviruses target the transcription (53) and surface expression of CD1d (26, 58) as a general mechanism for impeding iNKT cell recognition. Thus, future work focused on accurately quantifying the expression of CD1d during herpesvirus infection may yield important insights into the kinetics of iNKT cell recognition and lead to the identification of the lipid antigens(s) that are possibly triggered by herpesvirus infections. Such findings would support the involvement of iNKT cells in the control of herpesvirus infections and the hypothesis that herpesviruses downregulate the surface expression of CD1d to evade recognition by iNKT cells.

Lastly, we have shown EBV transformation suppresses the expression of CD1d and that the activation of the retinoic acid receptor pathway using AM580 re-establishes CD1d surface expression on LCL (53). This finding suggests that maintaining or restoring CD1d expression could improve anti-herpesvirus defenses and this approach could boost anti-viral defenses when combined with the concurrent administration of α-GalCer, or other iNKT cell agonists. Such strategies may enhance the priming of innate and adaptive immune responses to herpesviruses and promote the overall development of iNKT cell immunotherapies (111).

## Conflict of Interest Statement

The authors declare that the research was conducted in the absence of any commercial or financial relationships that could be construed as a potential conflict of interest.
